# Oxadiazole-Containing Macrocyclic Peptides Potentiate Azole Activity against Pathogenic *Candida* Species

**DOI:** 10.1128/mSphere.00256-20

**Published:** 2020-04-08

**Authors:** Nicole M. Revie, Nicole Robbins, Luke Whitesell, John R. Frost, Solomon D. Appavoo, Andrei K. Yudin, Leah E. Cowen

**Affiliations:** a Department of Molecular Genetics, University of Toronto, Toronto, Ontario, Canada; b Davenport Research Laboratories, Department of Chemistry, University of Toronto, Toronto, Ontario, Canada; University of Georgia

**Keywords:** *Candida*, *Candida albicans*, antifungal, azole, fluconazole, fungal pathogens, macrocycle, oxadiazole

## Abstract

Opportunistic pathogens of the genus *Candida* reign as the leading cause of mycotic disease and are associated with mortality rates greater than 40%, even with antifungal intervention. This is in part due to the limited arsenal of antifungals available to treat systemic fungal infections. Azoles have been the most widely deployed class of antifungal drug for decades and function by targeting the biosynthesis of ergosterol, a key component of the fungal cell membrane. However, their utility is compromised by their fungistatic nature, which favors the development of resistance. Combination therapy has the potential to confer enhanced efficacy as well as mitigate the evolution of resistance. Previously, we described the generation of structurally diverse macrocyclic peptides with a 1,3,4-oxadiazole and an endocyclic amine grafted within the peptide backbone. Importantly, this noncanonical backbone displayed high membrane permeability, an important attribute for compounds that need to permeate across the fungal cell wall and membrane in order to reach their intracellular target. Here, we explored the bioactivity of this novel chemical scaffold on its own and in combination with the azole fluconazole. Although few of the oxadiazole-containing macrocyclic peptides displayed activity against Candida albicans on their own, many increased the efficacy of fluconazole, resulting in a synergistic combination that was independent of efflux inhibition. Interestingly, these molecules also enhanced azole activity against several non-*albicans Candida* species, including the azole-resistant pathogens Candida glabrata and Candida auris. This work characterizes a novel chemical scaffold that possesses azole-potentiating activity against clinically important *Candida* species.

**IMPORTANCE** Fungal infections, such as those caused by pathogenic *Candida* species, pose a serious threat to human health. Treating these infections relies heavily on the use of azole antifungals; however, resistance to these drugs develops readily, demanding novel therapeutic strategies. This study characterized the antifungal activity of a series of molecules that possess unique chemical attributes and the ability to traverse cellular membranes. We observed that many of the compounds increased the activity of the azole fluconazole against Candida albicans, without blocking the action of drug efflux pumps. These molecules also increased the efficacy of azoles against other *Candida* species, including the emerging azole-resistant pathogen Candida auris. Thus, we describe a novel chemical scaffold with broad-spectrum bioactivity against clinically important fungal pathogens.

## Abstract (precis)

Fungal infections, such as those caused by pathogenic *Candida* species, pose a serious threat to human health. Treating these infections relies heavily on the use of azole antifungals; however, resistance to these drugs develops readily, demanding novel therapeutic strategies. This study characterized the antifungal activity of a series of molecules that possess unique chemical attributes and the ability to traverse cellular membranes. We observed that many of the compounds increased the activity of the azole fluconazole against Candida albicans, without blocking the action of drug efflux pumps. These molecules also increased the efficacy of azoles against other *Candida* species, including the emerging azole-resistant pathogen Candida auris. Thus, we describe a novel chemical scaffold with broad-spectrum bioactivity against clinically important fungal pathogens.

## OBSERVATION

Fungi have emerged as an important cause of human mortality worldwide, largely as a consequence of an expanding immunocompromised population (1). The opportunistic pathogen Candida albicans reigns as a leading cause of nosocomial infection and is associated with mortality rates greater than 40%, even with antifungal intervention (2). The azoles target the ergosterol biosynthetic enzyme lanosterol 14-α-demethylase. Although their impressive safety profile and oral bioavailability have led to widespread clinical use, their fungistatic mode of action and use as a prophylactic agent have rendered the azoles vulnerable to resistance development (3). A promising strategy for combating drug resistance is combination therapy, as it has the potential to confer enhanced efficacy as well as mitigate the evolution of resistance (4). Promising scaffolds to explore for antifungal combinations include complex macrocycles, which are structurally complex, and a common topology of natural products (5, 6). Several macrocyclic compounds, including tacrolimus, cyclosporine, beauvericin, and geldanamycin, display various degrees of antifungal activity on their own and enhance azole efficacy against fungal pathogens through distinct mechanisms (7–11). Previously, we described the generation of structurally diverse macrocyclic peptides with a 1,3,4-oxadiazole and an endocyclic amine grafted within the peptide backbone (12). The noncanonical backbone stabilized conformationally rigid structures that displayed high membrane permeability, and the pentapeptide fragment of the oxadiazole-containing macrocycles adopted stable β-turn conformations in solution (12). This property has been utilized to design macrocycles that inhibit β-turn-mediated protein-protein interactions (13). However, the bioactivity of these molecules against fungi has yet to be explored.

**FIG 1:**
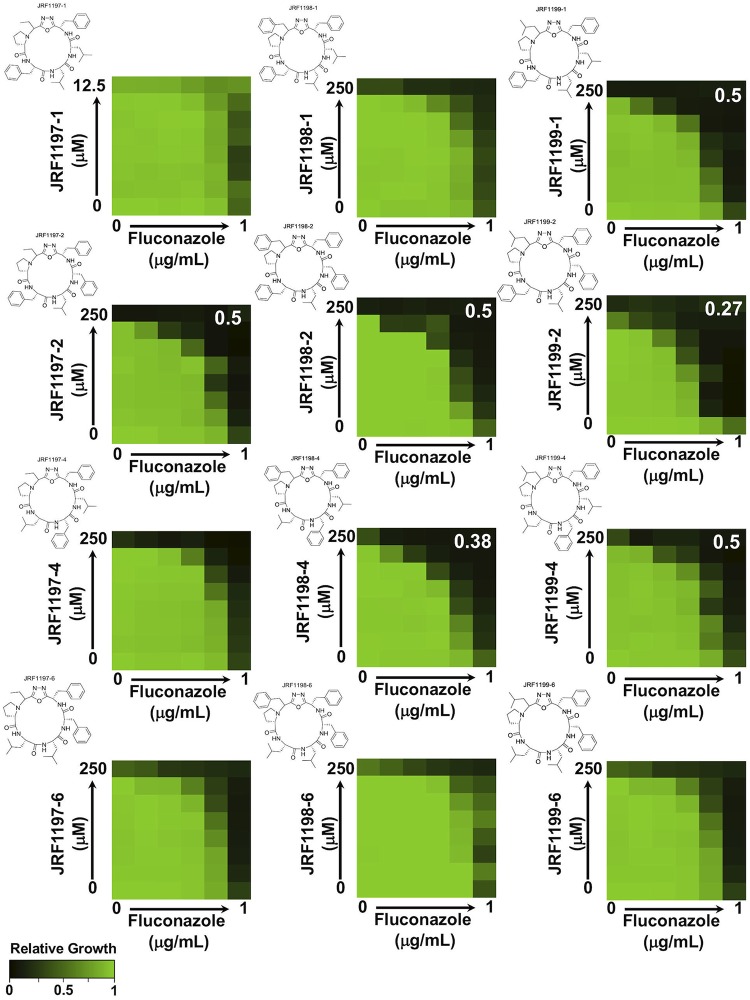
Select oxadiazole-containing macrocyclic peptides increase fluconazole efficacy against C. albicans. Checkerboard analysis of antifungal activity was performed with a combination of fluconazole and oxadiazole-containing macrocyclic peptides. C. albicans (SN95, CaLC239) was exposed to the indicated 2-fold serial dilutions of each compound for 24 h at 30°C in YPD medium. Optical densities were averaged for duplicate measurements and normalized relative to the no-drug control. Growth is quantitatively displayed by color, with green representing robust growth and black representing no growth (see color legend at bottom left). Chemical structures of the indicated macrocycles are provided to the upper left of each checkerboard. Compounds that display synergistic effects are indicated by an FICI value reported on the upper right of the checkerboard determined on the basis of calculations performed as described previously (15).

The aim of this study was to characterize the bioactivity of a collection of oxadiazole-containing macrocyclic peptides on their own and in combination with the azole fluconazole. To evaluate the activity of these compounds, we used a dose-response matrix (checkerboard) approach involving 2-fold dilution gradients of macrocycles, in combination with a 2-fold gradient of fluconazole, against a laboratory strain of C. albicans (SN95) (14). Although several macrocycles displayed single-agent activity at 250 μM, in general, the panel of compounds did not display strong bioactivity on their own against C. albicans in yeast extract-peptone-dextrose (YPD) medium at 24 h (Fig. 1). In contrast, several oxadiazole-containing macrocyclic peptides potentiated the efficacy of fluconazole against C. albicans. Compound interactions were quantified using the fractional inhibitory concentration (FIC) index (FICI), in which synergism is indicated by a FICI value of ≤0.5 (15). JRF1199-1, JRF1197-2, JRF1198-2, JRF1199-2, JRF1198-4, and JRF1199-4 all displayed a synergistic interaction with fluconazole (Fig. 1), highlighting that a phenylalanine residue in the C-terminal position of the peptide fragment is required for activity. In addition, active macrocycles had high levels of phenylalanine and leucine content, suggesting a potential interaction with a hydrophobic interface at the relevant molecular target. Next, we wanted to assess if the drug combination elicited a fungistatic or fungicidal effect. We performed checkerboard assays in the presence of fluconazole and our most potent oxadiazole-containing macrocyclic peptide, JRF1199-2, against C. albicans. After 48 h of incubation, cells were transferred onto drug-free medium and left to grow for 24 h to assess cell viability. At the highest concentrations of drugs tested, C. albicans was unable to recover, suggesting a fungicidal combination (see Fig. S1 in the supplemental material).

10.1128/mSphere.00256-20.1 FIG S1 JRF1199-2 in combination with fluconazole generates a fungicidal drug combination. Checkerboard assays were performed on a laboratory strain of C. albicans (SN95) in YPD incubated at 30°C for 48 h. Subsequently, 0.5 μl of culture was spotted onto YPD agar and incubated at 30°C for 24 h (shown to the right of the checkerboard heat map). Checkerboard data were analyzed as described for Fig. 1, and spots were imaged using a scanner. Download FIG S1, TIF file, 0.2 MB. Copyright © 2020 Revie et al. 2020 Revie et al. This content is distributed under the terms of the Creative Commons Attribution 4.0 International license.

**FIG 2:**
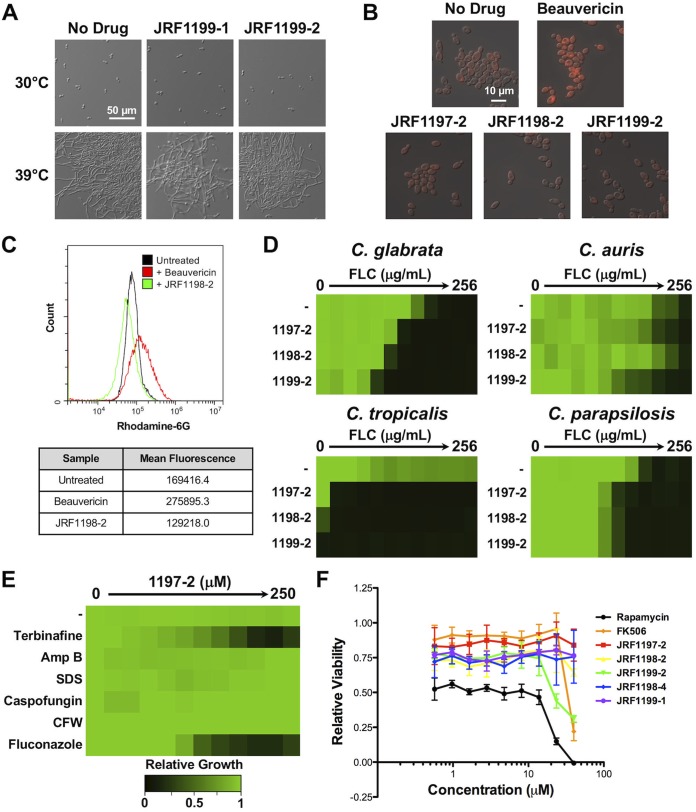
Oxadiazole-containing macrocyclic peptides do not impede C. albicans filamentation or multidrug efflux but do possess broad-spectrum ergosterol biosynthesis inhibitor-potentiating activity. (A) The effect of JRF1199-1 and JRF1199-2 on filamentation of C. albicans (SN95, CaLC239) was monitored after incubation in YPD medium at 30°C or 39°C for 4 h with shaking. Images were taken by differential interference contrast microscopy. Representative fields from micrographs obtained at the same magnification for all images are presented. (B) C. albicans (Caf2-1, CaLC2742) was grown in YPD medium at 30°C for 3 h with or without 125 μM macrocycle or 10 μg/ml of beauvericin. A concentration of 1 μg/ml of rhodamine-6G was added to the cultures for another 30 min at 30°C. Cells were washed twice with phosphate-buffered saline (PBS), followed by fluorescence microscopy to monitor rhodamine-6G accumulation in cells. (C) The fluorescence of untreated cells or of cells treated with beauvericin or JRF1198-2 was quantitated by flow cytometry. Assays were performed using 250 μl of culture per well, and fluorescence was measured using the FL2 (phycoerythrin) channel in a CytoFLEX flow cytometer (Beckman Coulter Inc.) with at least 20,000 events acquired per sample. Events were gated to capture at least 90% of the entire population analyzed, discarding clumps/cellular debris. Histograms representative of gated events are shown. (D) Fluconazole dose-response assays for diverse *Candida* species were conducted in YPD medium without (−) or with the indicated macrocycle (62.5 μM). Growth was measured by absorbance at 600 nm after 24 h (C. parapsilosis [CpLC573], C. auris [Ci 6684, CauLC5083], and C. glabrata [BG2, CgLC1002]) or after 48 h (C. tropicalis [CtLC573]) at 30°C. Optical densities were averaged for duplicate measurements and normalized relative to the no-drug control well for each strain. Growth is quantitatively displayed by color, with green representing robust growth and black representing no growth (see color legend). (E) Dose-response assays against a wild-type strain of C. albicans (SN95, CaLC239) were performed in YPD medium at 30°C, and absorbance at 600 nm was measured after 24 h as described for Fig. 1. These assays were performed with fixed subinhibitory concentrations of cell membrane/wall stressor as follows: terbinafine 0.3125 μg/ml, amphotericin B (Amp B) 0.24 μg/ml, sodium dodecyl sulfate (SDS) 0.0078% [mass/vol], caspofungin 0.03125 μg/ml, calcofluor white (CFW) 15 μg/ml, and fluconazole 0.25 μg/ml. (F) Macrocycle cytotoxicity profiling using RAW 264.7 murine macrophages was performed at 72 h, using quadruplicate wells and a standard resazurin dye reduction cell viability assay. The novel macrocycles showed modest cytotoxicity over a broad concentration range compared to control macrocycles rapamycin and tacrolimus (FK506).

Through a variety of mechanisms, macrocycles modulate a morphological transition between yeast and filamentous growth that is fundamental to the virulence of C. albicans (16, 17). Certain macrocycles have been reported to induce constitutive filamentous growth (18) or, alternatively, block filamentation upon exposure to an inducing cue (19). Thus, we examined the ability of representative oxadiazole-containing macrocyclic peptides to modulate C. albicans morphogenesis. When cells were grown in YPD at 30°C, C. albicans grew as yeast in the absence and presence of JRF1199-1 or JRF1199-2, two of the most highly bioactive macrocycles (Fig. 2A). Further, when cells were grown at the elevated temperature of 39°C, a condition that promotes filamentation, they underwent polarized growth in the absence and presence of either compound (Fig. 2A). Thus, oxadiazole-containing macrocyclic peptides do not modulate C. albicans filamentation.

Many macrocycles, including tacrolimus and beauvericin, potentiate azole activity in part due to their ability to inhibit multidrug transporters (11, 20, 21). Thus, we assessed whether the azole-potentiating activity of the oxadiazole-containing macrocyclic peptides was due to inhibition of efflux. To do so, we monitored intracellular accumulation of rhodamine-6G, a substrate of ABC transporters Cdr1 and Cdr2 (22), in C. albicans treated with compounds JRF1197-2, JRF1198-2, and JRF1199-2. These compounds were selected based on their bioactivity and structural diversity. Treatment with beauvericin (11) enhanced rhodamine-6G accumulation as visualized by microscopy and quantified using flow cytometry (Fig. 2B and C). However, none of the prioritized oxadiazole-containing macrocyclic peptides had a measurable effect on rhodamine-6G accumulation, suggesting that their capacity to enhance azole activity was independent of efflux.

To evaluate the activity of the oxadiazole-containing macrocyclic peptides against other fungal pathogens, we tested a panel of azole-resistant non-*albicans Candida* species, including C. glabrata, C. auris, C. tropicalis, and C. parapsilosis. Dose-response assays were conducted using standard protocols (14) with a 2-fold dilution series of fluconazole, without or with JRF1197-2, JRF1198-2, or JRF1199-2 at 62.5 μM, a concentration that enhanced azole activity against C. albicans (Fig. 1). In the absence of the oxadiazole-containing macrocyclic peptides, the fluconazole MICs for the strains ranged from 32 μg/ml to 256 μg/ml. All of the compounds increased the efficacy of fluconazole against C. glabrata and C. parapsilosis (Fig. 2D). JRF1197-2 and JRF1199-2 also displayed modest activity against the emerging pathogen C. auris (23). Surprisingly, these compounds also displayed activity against C. tropicalis at 62.5 μM in the absence of azole. These species-specific effects could be attributed to differences in compound permeability, efflux, or species-specific differences in a relevant cellular target(s).

Subsequently, we assessed if oxadiazole-containing macrocyclic peptides were capable of enhancing the activity of other antifungals and cellular stressors. The potentiating activity of one of the most potent molecules, JRF1197-2, was tested against the ergosterol biosynthetic inhibitor terbinafine, the cell membrane-targeting agents sodium dodecyl sulfate (SDS) and amphotericin B, and the cell wall-targeting agents calcofluor white and caspofungin. Testing was performed with a 2-fold gradient of JRF1197-2 in the absence or presence of a subinhibitory concentration of the cellular stressors. JRF1197-2 specifically potentiated the activity of the ergosterol biosynthetic inhibitors fluconazole and terbinafine but did not enhance the activity of the other agents tested (Fig. 2E).

Finally, we assessed mammalian cell cytotoxicity of the oxadiazole-containing macrocyclic peptides using RAW264.7 macrophages. The macrocyclic compounds tacrolimus (FK506) and rapamycin were used as structurally diverse control compounds that enhance azole activity against fungi but for which their clinical potential is impaired by their immunosuppressive and cytotoxic activities (24). Our prioritized oxadiazole-containing macrocyclic peptides displayed modest cytotoxicity after a 72-h exposure under standard assay conditions (25), with many compounds displaying no toxicity to mammalian cells at up to 40 μM (Fig. 2F). This was significant, as many oxadiazole-containing macrocyclic peptides potentiated azole activity at concentrations as low as 4 μM (Fig. 1).

This work characterizes a novel oxadiazole-containing macrocyclic peptide scaffold that possesses azole-potentiating activity against pathogenic *Candida* species. Future mechanistic studies will be useful in refining our understanding of the determinants contributing to the antifungal activity of azoles. The insights gained could enable additional chemical efforts to improve efficacy and selectivity, thereby contributing to the development of urgently needed therapies to combat fungal infections.
